# Biocompatible/Biodegradable Electrowetting on Dielectric Microfluidic Chips with Fluorinated CTA/PLGA

**DOI:** 10.3390/ma11081332

**Published:** 2018-08-01

**Authors:** Kaidi Zhang, Lei Chao, Jia Zhou

**Affiliations:** ASIC and System State Key Lab, School of Microelectronics, Fudan University, Shanghai 200433, China; zhangkaidi186@163.com (K.Z.); 14210720056@fudan.edu.cn (L.C.)

**Keywords:** fluorinated CTA/PLGA, EWOD, biocompatible, super-hydrophobicity

## Abstract

One of the major hurdles in the development of biocompatible/biodegradable EWOD (Electrowetting-on-dielectric) devices is the biocompatibility of the dielectric and hydrophobic layers. In this study, we address this problem by using reactive ion etching (RIE) to prepare a super-hydrophobic film combining fluorinated cellulose triacetate (CTA) and poly (lactic-co-glycolic acid) (PLGA). The contact angle (CA) of water droplets on the proposed material is about 160°. X-ray photoelectron spectroscopy (XPS) and atomic force microscopy (AFM) characterizations indicate that a slight increase in the surface roughness and the formation of CF_x_ (C-F or CF_2_) bonds are responsible for the super-hydrophobic nature of the film. Alternating Current (AC) static electrowetting and droplet transportation experiments evidence that contact angle hysteresis and contact line pinning are greatly reduced by impregnating the CTA/PLGA film with silicon oil. Therefore, this improved film could provide a biocompatible alternative to the typical Teflon^®^ or Cytop^®^ films as a dielectric and hydrophobic layer.

## 1. Introduction

In recent years, microfluidics has found numerous applications in the biomedical and environmental monitoring fields, for instance, in ecotoxicology [1,2], cell analysis [3,4,5,6], fertilization in vitro (IVF), cell culture [7,8], food detection [9] and soil analysis [10,11]. Due to its low-cost, versatility, and high integration [12,13], electrowetting-on-dielectric (EWOD) is at the forefront of digital microfluidics. EWOD technology can dispense, split, transport and mix droplets on a micro-scale platform under precise control [12,13,14,15,16]. It shows great potential to become one of the key components in future biological systems for sustainable development, such as real time analysis of water and soil quality [17,18], continuous monitoring for agricultural production [19,20,21] and point-of-care medical applications [22,23,24]. Nonetheless, a major issue in the implementation of these bio-systems is the lack of biocompatible/biodegradable EWODs [25,26].

A typical EWOD device consists of a substrate layer (glass or silicon), a driving electrodes layer (ITO, Al, Cu, etc.), a dielectric layer (Su-8, SiO_2_ etc.) and a hydrophobic/super-hydrophobic layer (e.g., Teflon^®^ or Cytop^®^) on top of it. When the voltage difference V is applied between a droplet and the electrode, the droplet surface becomes charged and is pulled towards the electrode, reducing the contact angle (CA) of the droplet. For a droplet of non-conducting liquid at mechanical equilibrium and at moderate electric voltage, this phenomenon can be described by the Lippmann-Young (L-Y) equation [27]:

(1) $$\cos\theta -\cos\theta_{0}=\frac{CV^{2}}{2\gamma }$$

where *θ* and *θ*_0_ are the contact angles with and without voltage *V* applied, respectively. *γ* is the surface tension between the liquid and the atmosphere. *C* (=*ε*_0_*ε_d_/d*) is the capacitance of the dielectric layer, where *ε_d_* and *d* are the relative dielectric constant and thickness of the dielectric layer, respectively. *ε*_0_ (8.85 × 10^−12^ F/m) is the vacuum permittivity.

To realize a fully biocompatible/biodegradable EWOD device, the glass/silicon substrate can replaced by polymethyl methacrylate (PMMA) or polydimethylsiloxane (PDMS) [25,28], and the electrodes can be made of biocompatible metals, such as Mn, Mg, etc. The critical point in the device is to fabricate a high-quality dielectric and hydrophobic/super-hydrophobic layer with biocompatible/biodegradable materials.

The main criteria when selecting an optimal material for biocompatible EWOD applications are the ease of processing this material on the substrate surface, the uniformity of the formed layer, the quality of the electrical insulation and the chemical stability of the material. A further requirement for a biodegradable material is to remain chemically stable over a certain timescale and degradable over a longer time. For these reasons, we favored cellulose triacetate (CTA) [29,30] and poly (lactic-co-glycolic acid) (PLGA) [31,32,33] over other choices such as ammonium persulfate (APS, synthesis) [34], poly(3-hydroxybutyrate-co-3-hydroxyhexanoate) (PHBHHx, synthesis) [35,36] and corn proteins (energy-efficient vapor deposition) [37].

In our recent study, we fabricated an EWOD device with a dielectric film composed of a CTA and PLGA mixture [38], while keeping the Teflon^®^/Cytop^®^ hydrophobic layer. In this paper, we address two problems of this previous embodiment: (i) we enhance the biocompatibility by eliminating the Teflon^®^/Cytop^®^ hydrophobic layer; and (ii) solve the DC asymmetric electrowetting effect [39,40,41,42,43,44] caused by a higher sensitivity to OH^-^ and other small ions. This is achieved by modifying the CTA/ PLGA dielectric film to cause it to be (super-) hydrophobic.

The (super-) hydrophobic nature of a dielectric film is mainly controlled by its chemical composition and surface geometry [45,46,47,48]. For instance, fluorocarbons CF_x_ (C-F or CF_2_) on the surface tend to lower the solid surface tension [29,49], while roughness at the micro/nano scale can magnify the hydrophobic nature of the same surface. Depending in the microstructure, there are two types of super-hydrophobicity in nature: the Cassie–Baxter state [50] (best exemplified by the lotus effect) and the Cassie-impregnating state [51] (also called the petal effect). Water droplets are unstable on a lotus leaf, whereas they are pinned to a rose petal, even when turned upside down [51,52]. Both the Cassie–Baxter and Cassie-impregnating state are metastable and turn into the Wenzel state under external interference, such as heat, vibration and electrical fields [53,54,55]. Once the droplet is in the Wenzel state, it is pinned to the surface and can hardly be driven by EWOD forces.

There are several methods to prevent or reverse this Cassie-to-Wenzel transition [56,57]. However, most of them involve external interference that can hardly be integrated into a biocompatible/biodegradable EWOD chip, and might be harmful to the human body. In addition, few of these methods are compatible with the EWOD manipulation of droplets. An option to prevent the Cassie-to-Wenzel transition for a water-based droplet is to infuse a hydrophobic lubricant that strongly wets the solid surface and is immiscible with water [56]. Silicon oil can be applied as the filler in surface pores [57]. This also contributes to reduce the actuation voltage by minimizing the contact angle hysteresis.

In this paper, we will first study the fluorination and microstructuring of the CTA/PLGA surface to fabricate biocompatible/biodegradable dielectric and super-hydrophobic layers for EWOD devices. The CF_4_/CHF_3_ reactive ion etching (RIE) process is then analyzed and optimized by etching rate calculations, X-ray photoelectron spectroscopy (XPS), and atomic force microscopy (AFM). The AC static electrowetting test was carried out in air and “air after oil” (substrate dried after impregnation with silicon oil). Eventually, the improved EWOD device will be tested for droplet transportation. Using the PLGA/CTA mixture as both the dielectric and hydrophobic layer involves much lower costs than the traditional materials, such as using SU-8 as dielectrics and Teflon as the hydrophobic layer. In addition, the fabrication process is compatible with the integrated circuits (IC) industry and is suitable for mass production in the future.

## 2. Materials and Methods 

### 2.1. Materials and Apparatus

The CTA pellets and the PLGA foam (lactide:glycolide = 75:25) were purchased from Sigma-Aldrich Co., Ltd. (St. Louis, MO, USA). Methylene chloride (MC) and *N*,*N*-demethylformamide (DMF) provided by Shanghai Chemical Reagent Co., Ltd. (Shanghai, China) were used as the solvents of CTA and PLGA, respectively. Low-viscosity silicone oil (5 mm^2^/s, 25 °C) was purchased from Sigma-Aldrich to be used as the medium in the measurements. The glass substrate coated with 130 nm of indium tin oxide (ITO) with a sheet resistance of about 15 Ω per square was purchased from Wesley technology Co., Ltd. (Foshan, Guangdong, China).

The spin-coater (WZ-400BZ-6NPP, Laurell Technologies Co., Ltd., North Wales, PA, USA) was used for the deposition of the dielectric layer. The hot plate and the oven (SmartLab HP-303DU, Strider Instrument & Application Co., Ltd., Shanghai, China) were used for post-baking and hard-baking, respectively. To modify the surface wettability of the dielectric layers, a reactive ion etcher (RIE-10NR, Samco International, Kyoto, Japan) was utilized.

The thickness of the film was measured by profilometer (Dektak XT, BRUKER, Karlsruhe, Germany). The surface elemental composition was analyzed by XPS (Thermo Scientific^TM^ K-Alpha^TM^, Thermo Fisher Scientific Co., Ltd., Waltham, MA, USA). The surface roughness was observed by AFM (Bruker DI D3100, BRUKER, Karlsruhe, Germany). The CA measurements were carried out by the droplet shape analyzer (DSA30, KRUSS, Hamburg, Germany). The driving signals for electrowetting and droplet manipulation were provided by the signal generator (FG503, MOTECH, Tainan, Taiwan) and the amplifier (HA-45, MOTECH, Tainan, Taiwan).

### 2.2. Preparation and Measurements

In the experiments, the ITO glass substrates were cleaned in acetone, ethanol and deionized (DI) water successively, and dried out with pure N_2_. The CTA pellets were dissolved in MC, producing a 1.2 wt. % solution, and the PLGA foam was dissolved in DMF, producing a 10 wt. % solution. These two solutions were mixed together with a ratio of MC/DMF (80/20 by volume). The mixture was spin-coated on the ITO glass substrate at 4000 rpm for 45 s. Then these samples were cured in an oven at 100 °C for 30 min. We repeated the coating and curing steps as many times as necessary to reach the desired thickness.

A CF_4_ plasma treatment had previously been applied to the surface of the CTA and PLGA separately, as in Table 1. It was found that the PLGA film was etched by CF_4_ [58] and became thinner by 100 nm (under 10 sccm, 50 W, 2.0 Pa CF_4_ plasma treatment), while the thickness of the CTA film increased slightly due to the deposition of CF_4_ instead of etching [29]. Both of the surfaces became hydrophobic after the RIE. 

After trials of different parameters, an optimized RIE workflow to introduce CF_x_ into the PLGA was defined in the four steps shown in Table 2. Each step has to be carried out successively on the dielectric coatings directly through CF_4_ plasma treatment under different gas flow rates and power levels. In the last step, CHF_3_ gas was used instead of CF_4_ to decrease the roughness of the modified surface. The thickness of the samples was measured during the process, and after each step the atomic composition of carbon/oxygen/fluorine (C/O/F) and their chemical bonds were characterized by XPS. In addition, the surface roughness was analyzed by AFM. The effects of each step are explained in Table 3.

**Figure 1:**
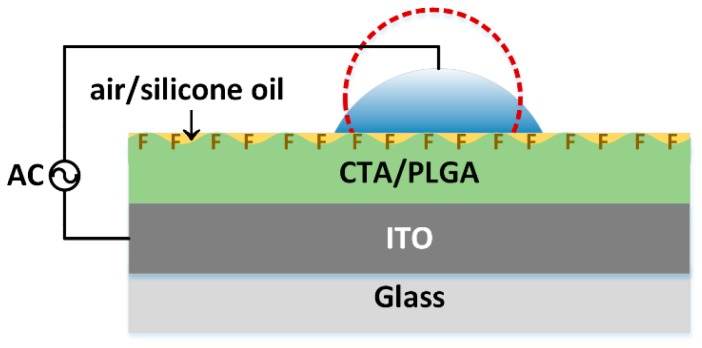
Schematics of CA measurement with the filler medium of air or silicone oil.

The CA measurements were performed on two types of substrates. Dry substrates “air” were tested directly after fabrication, whereas “air after oil” substrates were first immersed in silicone oil and left to dry naturally. A 6 μL Deionization (DI) water droplet was placed on the surface of “air” or “air after oil” and exposed to an alternating voltage (AC) between a conductive wire and the grounded ITO electrode, as shown in Figure 1. The shape of the droplet was captured and analyzed to obtain the CAs.

The droplet transportation test was carried out using an “air after oil” EWOD chip with patterned electrodes. AC voltage was applied to the electrodes with a certain sequence to manipulate the droplet. The process was captured and the relationship between the transporting velocity and the AC signal was obtained.

## 3. Results and Discussion

### 3.1. Etching Rate

The RIE was utilized to enhance the hydrophobicity of CTA/PLGA coatings. During this process, the PLGA was etched through a reaction with CF_4_ plasma. The etching rate of the mixed dielectrics was calculated.

**Figure 2:**
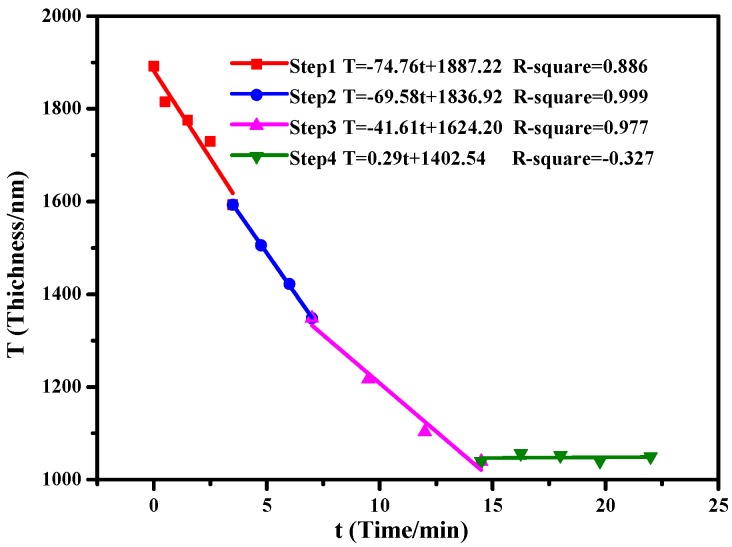
Change of dielectric thickness after RIE steps 1–4.

Figure 2 shows the etching rate under different flow and power rates, obtained by linear fitting. In steps 1–3, with the decrease of plasma power (from 100 to 50 W) and flow rate (from 30 to 10 sccm) of the CF_4_ gas supply, the etching rate was decreased from 74.76 nm/min in step 1 to 69.58 nm/min during step 2 and 41.61 nm/min in step 3. We believe this decreasing etching rate is due to the rarefaction of the PLGA as it reacts with the plasma. The thickness of the dielectrics showed almost no change (0.29 nm/min) when using CHF_3_ instead of CF_4_ in step 4, indicating the absence of etching. The surface roughness was reduced and the amount of fluorocarbons CF_x_ increased during the CHF_3_ gas treatment on the dielectric surface, which could also cause the thickness fluctuation. Finally, a 1.1 µm thick CTA/PLGA dielectric layer was obtained.

### 3.2. Mechanisms of the RIE Process

During the RIE process, fluorine atoms react with the mixture surface to form fluorocarbons. By this means, the surface tension was decreased to enhance the hydrophobicity of CTA/PLGA dielectrics.

Table 4 and Table 5 show the XPS narrow scan for C/O/F atoms after each step of the process. Prior to the treatment, we hardly detected any presence of fluorine on the sample. After step 1, the ratio of F atoms increased to 29.7%. The results illustrated that fluorine was introduce into the mixture surface in step 1, and then decreased slightly to 26.2% in step 2, and then increased continually to 36.4% in step 3. Eventually, the fraction of F atoms increased slightly to 40.8% in step 4. During the RIE process, there was a sharp rise in F atoms during CF_4_ gas treatment, and then the ratio of F atoms was maintained with the CHF_3_ gas treatment. For more details, the chemical nature of the F bonds is reported in Table 5. The majority (62.3%) of F atoms formed C-F bonds with C atoms, and about 10.4% formed CF_2_ bonds. Since the samples were preserved in aluminum foils, AlFx was also observed in the XPS analysis.

**Figure 3:**
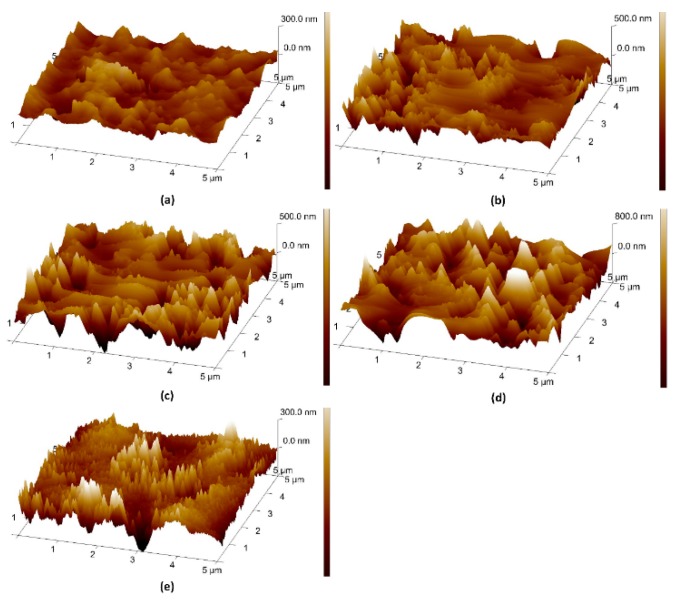
AFM scanning images of (**a**) step 1, (**b**) step 2, (**c**) step 3, (**d**) step 4 and (**e**) untreated.

The effect of surface roughness on the wetting property was investigated. Figure 3 shows the AFM scans of these samples. The roughness of the surfaces is detailed in Table 6. The surface roughness was 43.70 nm before RIE (Figure 3e). The roughness increased to 97.90 nm after etching for 3.5 min (Figure 3a), which led to the pinning of the droplet on the surface. To mitigate this effect, the flow rate and power were decreased gradually; the surface roughness increased by 27 nm after 3.5 min etching (Figure 3b) and 84 nm after 7 min etching (Figure 3c). Eventually, the surface was treated with CHF_3_ to make it smoother (Figure 3d). The final surface roughness was about 66.5 nm. Millimetric droplets (volume > 10 μL) were able to easily slide down the surface.

**Figure 4:**
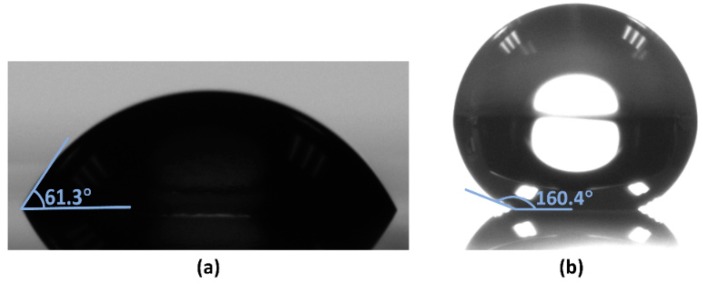
DI water droplet on the mixture film (**a**) untreated and (**b**) fluorinated by RIE.

After the ion etching, the CA of the deionized water droplets increased to 160.4°, compared to 61.3° without fluorinated treatment, which indicates that the surface was super-hydrophobic, as shown in Figure 4.

### 3.3. AC Static Electrowetting Test

**Figure 5:**
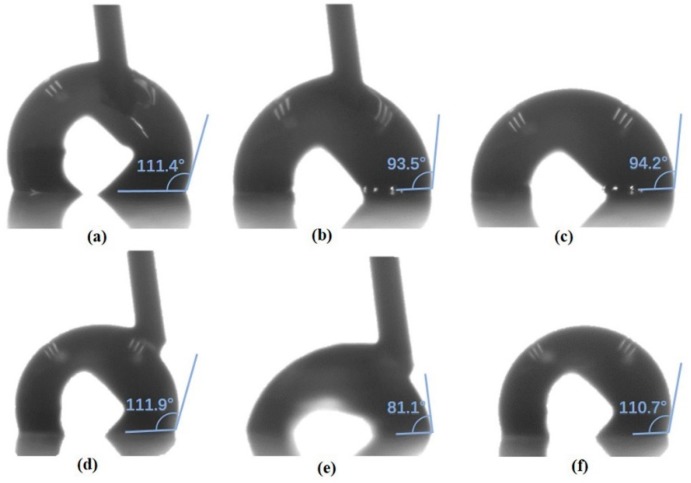
AC static electrowetting test in the air under (**a**) AC off (**b**) 44V_rms_ (**c**) AC off again, and in the “air after oil” under (**d**) AC off (**e**) 44V_rms_ (**f**) AC off again.

To examine the applicability of the fluorinated CTA/PLGA in EWOD, we first carried out an AC static electrowetting test. Considering the thickness and roughness of the processed mixture coatings, the filler medium in the pores was important [57]. Thus, air and silicone oil were chosen as the medium for this experiment. Figure 5 illustrates the results. When tested in the air directly, the droplet was pinned on the surface even without AC voltage, as seen in Figure 5a. Bubbles emerged on the interface in Figure 5b, which indicates the dielectric breakdown of the 1.1 μm layer under 44V_rms_. When the voltage was off, the CA could not be recovered (Figure 5c). In contrast, in the “air after oil” configuration, the droplet moved smoothly on the surface (Figure 5d). With the help of residual oil on the surface, the CA of the DI water droplet decreased (Figure 5e) and reverted to its original value (Figure 5f) when the voltage was on and off. This suggests that the oil filling not only reduces the contact angle hysteresis by smoothing out the chemical imperfections of the surface, but also prevents dielectric breakdown by accumulating some electric charge.

### 3.4. Droplet Transportation Test

The CTA/PLGA layer was applied to an EWOD transportation chip to verify its ability to act as the dielectric and hydrophobic layer simultaneously. The size of the transporting electrode was 3 mm × 1 mm, with a gap of 30 µm between electrodes. Following the patterning of the electrodes, the EWOD chip was fabricated by the spin coating of CTA/PLGA, RIE and the “air after oil” process, as described above. When a 1 kHz AC voltage was applied to the successive electrodes, the 15-µL droplet moved up and down.

**Figure 6:**
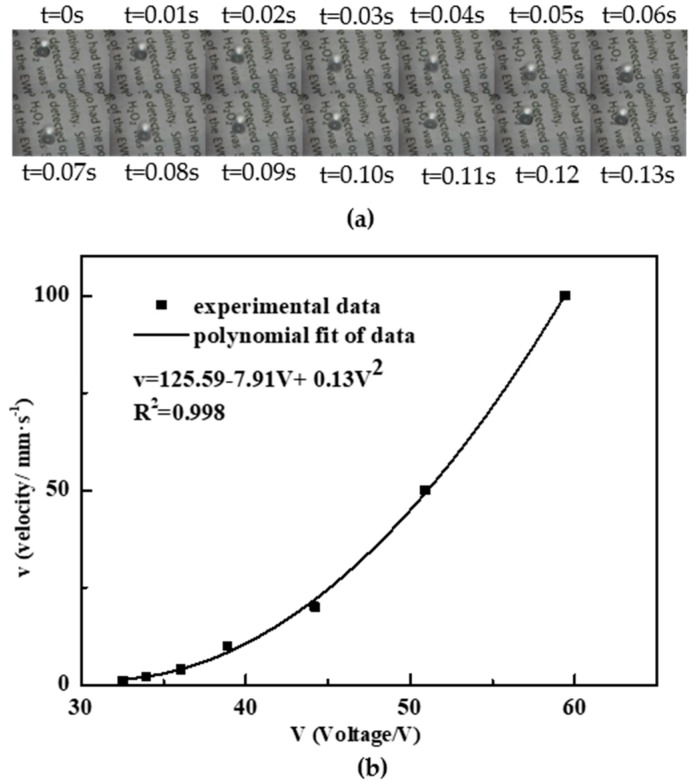
Electrowetting transportation test: (**a**) images of droplet transportation; (**b**) droplet velocity versus voltage (r.m.s).

Video (see in Video S1) and images (see Figure 6a) of the process of droplet transportation was recorded. The relationship of the droplet velocity versus voltage (rms) is shown in Figure 6b. Depending on the driving voltage, we achieved droplet velocities from 1 mm/s (30 Vrms) up to 100 mm/s (60 Vrms). The polynomial curve fitting shows that the droplet velocity can be factored into (V − 30)^2^ within 2% accuracy, indicating an actuation threshold of 30 V, which agreed well with the theoretical properties and practical conclusions of common EWOD chips [60].

## 4. Conclusions

In this paper, we replaced the hydrophobic Teflon^®^ or Cytop^®^ layer of EWOD systems with a completely biocompatible/biodegradable layer. Using RIE supplied with CF_4_ and CHF_3_ gas under different processing parameters, the wettability of the CTA/PLGA mixture surface became super-hydrophobic (CA = 160.4°). XPS and AFM analysis showed that fluorine atoms were introduced onto the surface to form C-F and CF_2_ bonds. CHF_3_ was added to reduce the roughness and protect the fluorocarbons on the surface. By this means, a 1.1 μm super-hydrophobic dielectric layer was obtained with about 66.50 nm roughness. The “air after oil” modified surface prevented the pinning of DI water droplets on the surface and protected the dielectrics from breakdown. These digital microfluidic chips showed their suitability and high performance for applications involving bio-systems for sustainable development. The corresponding practical applications will be the subject of future studies.

## Figures and Tables

**Table 1:** Effects of the CF_4_ treatment on CTA and PLGA separately.

Material	Property	CF_4_ Treatment
CTA	Thickness	Increased a little
	Hydrophobicity	Increased
PLGA	Thickness	Decreased
	Hydrophobicity	Increased

**Table 2:** RIE workflow for the surface modification of a CTA/PLGA dielectric layer.

Step	Gas	Flow Rate (sccm)	Pressure (Pa)	Power (W)	Estimated Bias Voltage (V)	Time (min)
1	CF_4_	30	2.0	100	50	3.5
2	CF_4_	30	2.0	50	25	3.5
3	CF_4_	10	2.0	50	25	7.5
4	CHF_3_	20	2.0	50	25	7.5

**Table 3:** Effects of each RIE step.

Step	Effects
1	Introduces F atoms to form CF_x_ bonds. The roughness increases sharply, and the etching rate is high. Therefore, the time should not be too long [59].
2	Same as step 1 with slower increase of roughness and decreasing etching rate.
3	Same as step 1 and 2 but even slower
4	Reduces the roughness while keeping the CFx on the surface

**Table 4:** XPS spectra analysis of the C/F/O ratio (%).

Step	C	O	F
Step 1	51.4	18.8	29.7
Step 2	52.3	21.5	26.2
Step 3	45.3	18.3	36.4
Step 4	50.3	9.0	40.8
Untreated	59.9	40.1	N.D.

**Table 5:** XPS spectra analysis of fluorine bonding ratio (%).

Step	F1s (AlF_x_)	F1s (C-F)	F1s (CF_2_)
Step 1	37.6	55.0	7.4
Step 2	26.3	65.8	7.9
Step 3	32.9	57.2	10.0
Step 4	27.3	62.3	10.4
Untreated	N.D.	N.D.	N.D.

**Table 6:** Surface roughness variation during the RIE process.

Step	R_q_ (nm)
Step 1	97.90
Step 2	124.00
Step 3	208.00
Step 4	66.50
Untreated	43.70

## Supplementary Materials

The following are available online at http://www.mdpi.com/1996-1944/11/8/1332/s1, Video S1: Droplet transporting on PLGA/CTA.
